# Flagellin-dependent TLR5/caveolin-1 as a promising immune activator in immunosenescence

**DOI:** 10.1111/acel.12383

**Published:** 2015-07-30

**Authors:** Jae Sung Lim, Kim Cuc Thi Nguyen, Chung Truong Nguyen, Ik-Soon Jang, Jung Min Han, Claire Fabian, Shee Eun Lee, Joon Haeng Rhee, Kyung A Cho

**Affiliations:** 1 Department of Biochemistry, Chonnam National University Medical SchoolGwangju, 501-746, South Korea; 2 Center for Creative Biomedical Scientists, Chonnam National University Medical SchoolGwangju, 501-746, South Korea; 3 Clinical Vaccine R&D Center, Chonnam National University Hwasun Hospital160 Ilsim-Ri, Hwasun-gun, Jeonnam, 519-809, South Korea; 4 Division of Life Science, Korea Basic Science InstituteDaejeon, 305-333, South Korea; 5 Department of Integrated OMICS for Biomedical Science, Yonsei UniversitySeoul, 120-749, South Korea; 6 College of Pharmacy, Yonsei UniversityIncheon, 406-840, South Korea; 7 Department of Immunology, Fraunhofer Institute for Cell Therapy and Immunology (IZI), University of Leipzig04103, Leipzig, Germany; 8 Translational Center for Regenerative Medicine (TRM), University of Leipzig04103, Leipzig, Germany; 9 Dental Science Research Institute, School of Dentistry, Chonnam National UniversityGwangju, 500-757, South Korea; 10 Department of Microbiology, Chonnam National University Medical SchoolGwangju, 501-746, South Korea; 11 Research Institute of Medical Sciences, Chonnam National University Medical SchoolGwangju, 501-746, South Korea

**Keywords:** immunosenescence, vaccine adjuvant, TLR5, flagellin, caveolin-1

## Abstract

The age-associated decline of immune responses causes high susceptibility to infections and reduced vaccine efficacy in the elderly. However, the mechanisms underlying age-related deficits are unclear. Here, we found that the expression and signaling of flagellin (FlaB)-dependent Toll-like receptor 5 (TLR5), unlike the other TLRs, were well maintained in old macrophages, similar to young macrophages. The expression and activation of TLR5/MyD88, but not TLR4, were sensitively regulated by the upregulation of caveolin-1 in old macrophages through direct interaction. This interaction was also confirmed using macrophages from caveolin-1 or MyD88 knockout mice. Because TLR5 and caveolin-1 were well expressed in major old tissues including lung, skin, intestine, and spleen, we analyzed in vivo immune responses via a vaccine platform with FlaB as a mucosal adjuvant for the pneumococcal surface protein A (PspA) against *Streptococcus pneumoniae* infection in young and aged mice. The FlaB-PspA fusion protein induced a significantly higher level of PspA-specific IgG and IgA responses and demonstrated a high protective efficacy against a lethal challenge with live *S. pneumoniae* in aged mice. These results suggest that caveolin-1/TLR5 signaling plays a key role in age-associated innate immune responses and that FlaB-PspA stimulation of TLR5 may be a new strategy for a mucosal vaccine adjuvant against pneumococcal infection in the elderly.

## Introduction

Elderly individuals exhibit a decreased ability to resist infectious diseases and generate robust, protective immune responses against vaccine-preventable diseases, including influenza and pneumonia (Boraschi *et al*., 2010; Goronzy & Weyand, 2013). Although the T- and B-lymphocyte compartments of the adaptive immune system are known to progressively deteriorate with advancing age (Geiger & Rudolph, 2009; Palmer, 2013), the impact of aging on the innate immune system remains unclear.

Toll-like receptors (TLRs) play a crucial role as pattern recognition receptors in signaling danger and initiating the host innate responses. Among the TLRs, TLR5 has been newly considered as a promising candidate adjuvant for vaccines and immunotherapy (Faham & Altin, 2010; Mizel & Bates, 2010; Nguyen *et al*., 2013). TLR5 is stimulated by flagellin, the major structural component of bacterial flagella. Both human TLR5 and mouse TLR5 recognize similar molecular determinants of flagellin from various bacteria.

Many pathogenic bacteria use flagella to establish their niche at the surface of mucosal tissues. The sensing of flagellin by TLR5 may act as an early detection system that triggers a rapid host response (Hughes & Galan, 2002; Ramos *et al*., 2004). The TLR5 cascade depends exclusively on the TIR adaptor molecules MyD88, activating nuclear factor (NF)-kB and mitogen-activated protein kinase (MAPK) pathways (i.e., p38, JNK, and ERK1/2) that regulate the transcription of genes encoding immune mediators (Berin *et al*., 2002; Zeng *et al*., 2003). TLR expression is reportedly decreased in the immune cells of elderly human donors and aged mice (Panda *et al*., 2010; Shaw *et al*., 2013). The aging-dependent reduction in TLR1/4s might affect the decreased antibody responses to influenza vaccination in old donors (Liu *et al*., 2011). TLR2 signaling was also diminished in old alveolar macrophages and not fully effective against bacterial infection (Rottinghaus *et al*., 2010; Boyd *et al*., 2012). Thus, reduced levels of certain TLRs and diminished TLR signaling likely contribute to the impaired immune responses and the increased mobility and mortality that have been noted in elderly populations (Shaw *et al*., 2010, 2011).

Caveolin-1 is a structural protein of caveolae, which has specialized membrane microdomains, and has been implicated as a significant regulator in various physiological phenotypes, especially cellular senescence (Cho *et al*., 2003, 2004; Lim *et al*., 2009, 2010). Many signaling proteins interact with caveolin-1 through scaffolding domains, which regulates their activity. In senescence, upregulated caveolin-1 inhibits cellular growth through direct binding with growth factor receptors, leading to the senescence-associated growth arrest (Cho *et al*., 2003). Recently, caveolae/caveolin-1 has been identified in all types of immune cells and their expression and distribution might be dependent on the activation and maturation state of the cell (Harris *et al*., 2002; Medina *et al*., 2006). Caveolae also directly interact with outer pathogens, such as FimH-expressing *Escherichia coli*, mycobacteria, Cholera toxin, Simian virus 40, and human immunodeficiency virus (Lencer *et al*., 1999; Shin *et al*., 2000; Norkin *et al*., 2002; Zaas *et al*., 2005; Wang *et al*., 2010). Furthermore, surface receptors and signaling molecules are associated with caveolae and caveolae-like high-density lipid microdomains. BCR and CD19/21 in B cells translocate to lipid rafts after binding to complement-tagged antigens (Cherukuri *et al*., 2001), and TCR in Jurkat T cells colocalizes with cholera toxin B (Janes *et al*., 1999). However, the relationship between caveolin-1 and TLRs in innate immune systems is not well understood.

In this study, we demonstrated that FlaB-dependent TLR5 signaling and cytokine production were remarkably maintained in the macrophages of aged mice. Interestingly, this well-maintained TLR5 expression and signaling were tightly regulated by interactions with caveolin-1 and was elevated in old macrophages and in old tissues. Finally, through an *in vivo* vaccine platform using antigens with flagellin, we demonstrated a high efficacy of immune responses by flagellin-TLR5 activation and successful protection against *S. pneumonia* infection in old mice.

## Results

### Aging-dependent differential activation of TLRs in innate immune cells

**Fig 1:**
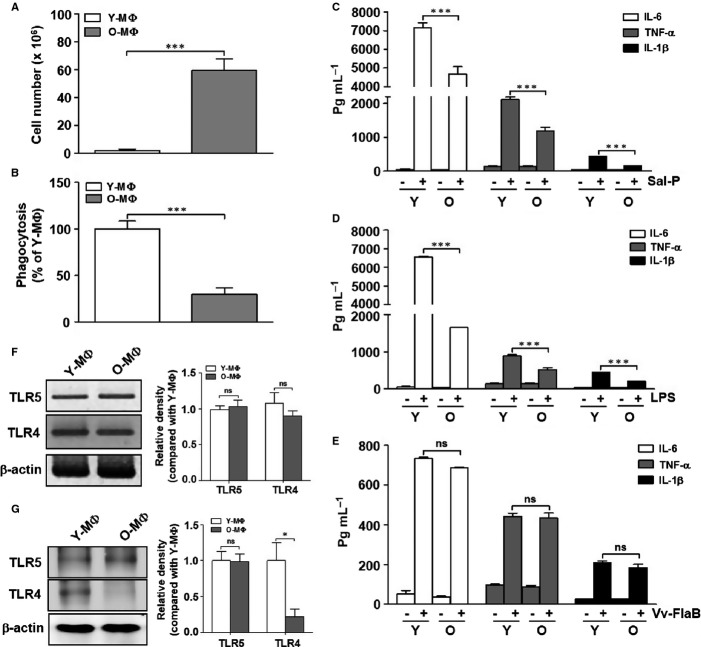
Age-associated functional alterations in peritoneal macrophages. Peritoneal macrophages were isolated from young (8 weeks old, *n* = 4∼5 mice) and aged mice (24 months old, *n* = 4∼5 mice). (A) The total cell numbers were counted and are represented by a graph. (B) Phagocytosis of live *Salmonella* into the young (Y-MΦ) and aged macrophages (O-MΦ) was quantified. The phagocytic capacity of the aged macrophages was expressed as values relative to the phagocytosis by the young macrophages. (C–E) The cells were stimulated with *Salmonella* proteins (C) (Sal-P, 10 μg/mL), (D) LPS (5 μg/mL), and (E) Vv-FlaB (100 ng/mL) for 12 h. Culture supernatants were collected and analyzed by ELISA for IL-6, TNF-α, and IL-1β cytokine secretion. (F) The mRNA levels of TLR4 and TLR5 in the young and aged macrophages were determined by RT-PCR. (G) The protein expression levels of TLR4 and TLR5 in the young and aged macrophages were determined by Western blotting with anti-TLR5 and anti-TLR4 antibodies. β-Actin was used as an internal control. The relative density of mRNA and proteins were normalized with β-actin and represented by the quantitative graphs, and differences between each compared group were analyzed by Mann–Whitney U-test. The data are based on five independent experiments. *P <* *0.05; ***P <* *0.001; ns, not significant.

To elucidate the functional changes in macrophages with advancing age, we isolated peritoneal macrophages from young (6–8 weeks old) and aged (>24 months old) C57BL/6 mice and compared their numbers and phagocytic activities. We obtained greater numbers of macrophages from the aged mice than from the young mice (Fig.1A). However, the phagocytic activity was significantly decreased in the macrophages from the aged mice (Fig.1B). These results suggest that aged mice may produce greater numbers of macrophages to compensate for their decreased function.

Next, we examined the levels of ligand-specific TLR-dependent proinflammatory cytokines, including IL-1β, IL-6 and TNF**-**α, after treatment with *Salmonella* extracts (Sal-P), which can induce the activation of several types of TLRs; LPS activated TLR4, and FlaB activated TLR5 in the macrophages from young and aged mice. The macrophages from the aged mice responded to stimulation with Sal-P and LPS, but the secretion of proinflammatory cytokines in the aged mice was lower than that in the young mice (Fig.1C and D). It is established that the splenic and thioglycollate-elicited peritoneal macrophages from aged mice have a lower surface expression of TLR4 than those from young mice (Renshaw *et al*., 2002). Notably, FlaB-dependent proinflammatory cytokine production was similarly induced in the macrophages from both the young and aged mice (Fig.1E). To elucidate the FlaB-dependent TLR5 activation in aging, we evaluated the expression of TLR5, with TLR4 as a negative control, in the macrophages from young and aged mice. The mRNA levels of TLR4 and TLR5 by RT–PCR did not differ between the young and aged macrophages (Fig.1F), whereas the TLR4 protein expression was significantly reduced in the aged macrophages, as similarly reported in other studies. TLR5 was well expressed in both young and aged macrophages (Fig.1G). Based on these results, we hypothesize that unlike other TLR pathways, the TLR5 expression and signaling pathway may be well preserved in the innate immune systems during aging.

### TLR5 expression was regulated by caveolin-1 via direct interactions in old macrophages

**Fig 2:**
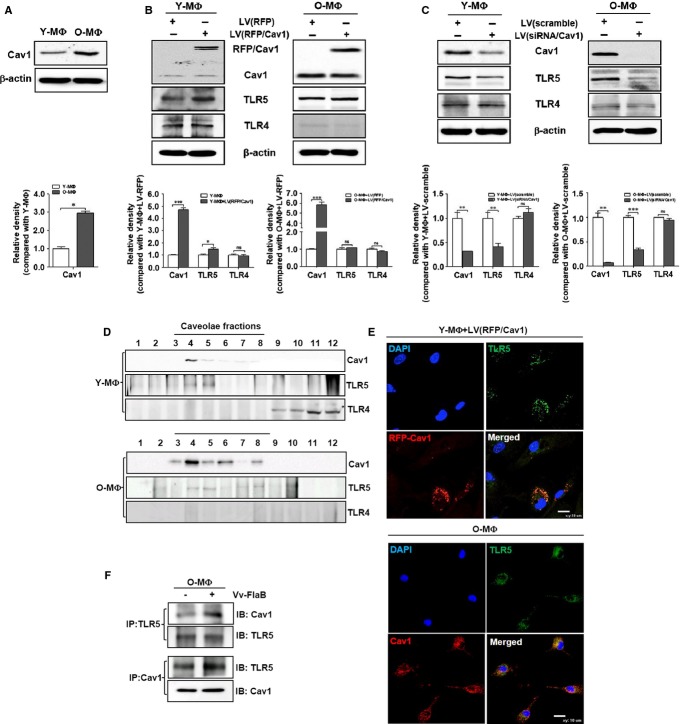
Caveolin-1-dependent TLR5 expression and localization. Peritoneal macrophages were isolated from young (*n* = 4∼5 mice) and aged mice (*n* = 4∼5 mice). (A) The basal protein expressions of caveolin-1 in the young and aged macrophages, the expression of Cav1 and TLRs in young and aged macrophages after lentiviral infection (B) carrying RFP-caveolin-1 in young (Y-MΦ) and aged macrophages (O-MΦ) or (C) carrying caveolin-1 targeting siRNA in Y-MΦ and O-MΦ were determined by Western blotting with anti-Cav1, anti-TLR5, and anti-TLR4 antibodies. β-Actin was used as an internal control. To elucidate the co-localization of TLR5 in caveolae, (D) caveolae-rich membrane domains were isolated from young and aged macrophages and detected by Western blotting with anti-Cav1, anti-TLR5, and anti-TLR4 antibodies. (E) RFP-caveolin-1 overexpressed young macrophages and aged macrophages were immunostained with anti-Cav1 (O-MΦ, red) or anti-TLR5 (green) antibody. Nucleus was stained by DAPI (blue), and all cells were analyzed by confocal microscopy. (F) After Vv-FlaB stimulation, the cell lysates were immunoprecipitated with anti-Cav1 or anti-TLR5 antibodies and then immunoblotted with anti-TLR5 and anti-Cav1 antibodies, respectively. The relative density of proteins was normalized with β-actin and represented by the graphs. Differences between each compared group were analyzed by Mann–Whitney U-test. The data are based on five independent experiments (A–C) or three independent experiments (D–E). *P <* *0.05; **P <* *0.01; ***P <* *0.001; ns, not significant. Cav1, caveolin-1.

Because we previously observed that caveolin-1 is highly increased in various immune-related, old organs, including the spleen and lymph node (data not shown), we studied the relationship between TLR5 and caveolin-1 in macrophages. When we compared the expression of caveolin-1 by Western blotting in macrophages from young and old mice, caveolin-1 was significantly increased in the old macrophages (Fig.2A). Next, whether the expression of TLR5 is regulated by caveolin-1 expression, we tested the TLR4/5 expression with presence or absence of caveolin-1 in young or aged macrophages. While overexpression of RFP-conjugated caveolin-1 in young macrophages led to increase TLR5, it did not affect to TLR5 expression in aged macrophages (Fig.2B). The expression of TLR5 was remarkably decreased by the downregulation of caveolin-1 by its targeting siRNA in both young and aged macrophages but not TLR4 (Fig.2C). These results reveal that the expression of TLR5, unlike TLR4, was sensitively affected by caveolin-1 expression. To clarify the relationship between TLR5 and caveolin-1, we examined their localization in young and aged macrophages. First, we isolated the caveolae-rich membrane domain from young and aged macrophages using sucrose-gradient ultracentrifugation (described in the Materials and methods section). The TLR5 and caveolin-1 were well detected in caveolae-rich fractions (from fraction No. 4 to fraction No. 8) from both young and aged macrophages. However, TLR4 was only detected in noncaveolae fractions (from No. 9 to fraction No. 12) from young macrophages (Fig.2D). This co-localization was also confirmed by immunofluorescence staining. TLR5 was clearly co-localized with RFP-caveolin-1 in young or endogenous caveolin-1 in aged macrophages (Fig.2E). Because the expression of TLR5 and caveolin-1 was increased by FlaB treatment (data not shown), we determined their direct interaction in old macrophages. After FlaB treatment in old macrophages, the cell lysates were pulled down with anticaveolin-1 or anti-TLR5 antibodies and then analyzed by Western blotting TLR5 was interacted with caveolin-1 without FlaB, and their interaction was remarkably enhanced by the FlaB treatment in old macrophages (Fig.2F).

### Caveolin-1 mediates TLR5/Myd88 signaling

**Fig 3:**
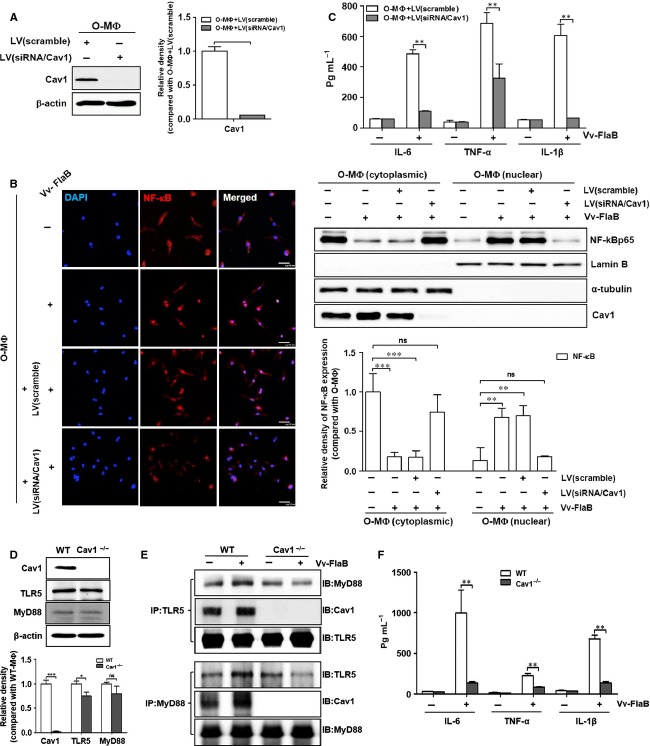
Caveolin-1 mediates TLR5/MyD88 signaling. To clarify the role of caveolin-1 in TLR5 signaling, caveolin-1 expression was knocked down using a lentivirus carrying caveolin-1-targeted siRNA in peritoneal macrophages from aged mice (*n *= 4∼5 mice). (A) The protein expression of caveolin-1 was analyzed by Western blotting. (B) The infected cells were treated with Vv-FlaB and then stained with an anti-NF-κB antibody (red) and DAPI (blue). These cells were fractionated with cytoplasmic and nucleus and determined by Western blotting with anti-NF-κB, anti-α-tubulin (cytoplasm marker), antilamin B (nucleus marker), and anti-Cav1 antibodies. (C) After stimulation with Sal-P, LPS, and Vv-FlaB for 12 h, the culture supernatants were collected and analyzed by ELISA for IL-6, TNF-α, and IL-1β cytokine secretion, and their interactions were confirmed in peritoneal macrophages isolated from Cav1^−/−^ and WT mice (*n* = 4∼5 mice per each group). (D) The protein expression levels in each cell were analyzed by Western blotting. After stimulation for 12 h with or without Vv-FlaB, (E) the interactions of TLR5 and MyD88 were analyzed by immunoprecipitation assay with anti-TLR5 or anti-MyD88 antibodies, respectively, and immunoblotted with anti-MyD88, anti-TLR5, and anti-Cav1 antibodies. (F) After 12 h of stimulation with Vv-FlaB, the culture supernatants were collected and analyzed by ELISA for IL-6, TNF-α, and IL-1β cytokine secretion. The relative density of proteins was normalized with β-actin (Fig.3A and 3D) or α-tubulin and lamin B (Fig.3B) and represented by the graphs. Differences between each compared group were analyzed by Mann–Whitney *U*-test. The data were based on three independent experiments (A–C) or five independent experiments (D-F). *P <* *0.05; **P <* *0.01; ***P <* *0.001; ns, not significant. WT, wild-type mice; Cav1^−/−^, caveolin-1 knockout mice.

To elucidate the role of caveolin-1 in TLR5 signaling in old macrophages, we examined NF-kB translocation and pro-inflammatory cytokine secretion after caveolin-1 downregulation by its targeted siRNA treatment. After analyzing the caveolin-1 expression by siRNA treatment (Fig.3A), we used confocal microscopy to detect NF-kB translocation from the cytoplasm to the nucleus in old macrophages. Whereas NF-kB successfully entered the nuclei by FlaB treatment in old and control siRNA-treated old macrophages, treatment with caveolin-1 siRNA inhibited this translocation (Fig.3B, left panel). The translocation of NF-kB was also repeated by subcellular fractionation. NF-kB was well detected in nuclear fraction from cytoplasmic fractions after stimulation of FlaB in aged- and siRNA/control-treated macrophages. However, it failed to induce this translocation of NF-kB in caveolin-1-down-regulated aged macrophages (Fig.3B right panel). NF-kB-dependent proinflammatory cytokine production was also decreased by treatment with caveolin-1 siRNA compared with the control (Fig.3C). These results suggest that upregulating caveolin-1 might mediate TLR5 signaling via a direct interaction in old macrophages. To clarify the relationship between caveolin-1 and TLR5 signaling, we analyzed the direct interaction of TLR5/MyD88 with caveolin-1 using primary macrophages from caveolin-1 (Cav1^−/−^) or MyD88 (Myd88^−/−^) knockout mice. The TLR5 expression was slightly decreased in macrophages from Cav1^−/−^ mice, but not from MyD88 mice (Fig.3D and Fig. S1A). A FlaB-dependent increase in TLR5 and caveolin-1 was not detected in the Cav1^−/−^ mice (data not shown). Furthermore, the interaction of TLR5 with MyD88 was not increased by FlaB treatment in the Cav1^−/−^ mice, leading to reduced pro-inflammatory cytokine production, compared with the WT (Fig.3E and F). We also confirmed these results using macrophages from the Myd88^−/−^ mice. FlaB-dependent expression and interaction of TLR5/caveolin-1 was not increased in macrophages from the Myd88^−/−^ mice (Fig. S1A and B). IL-6 production was not absent in macrophages from the Myd88^−/−^ mice compared with macrophages from the WT (Fig. S1C). These results indicate that caveolin-1 might form a triple complex with TLR5/MyD88 and play a crucial role in FlaB-dependent TLR5 signaling in macrophages.

### Expression of TLR4/5 and caveolin-1 between young and aged tissues

**Fig 4:**
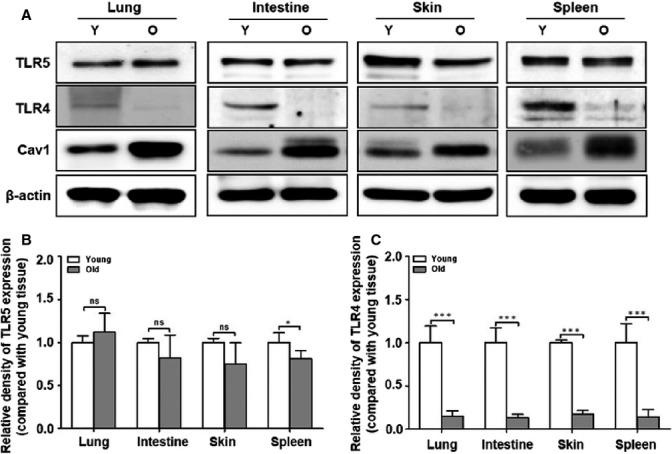
Expression levels of TLRs in various tissues from young and old mice. Tissues were isolated from young and aged mice. (A) The protein expression in the various tissues from young and aged mice was analyzed by Western blotting with anti-TLR5, anti-TLR4, anti-Cav1, and β-actin antibodies. The quantitative results of (B) TLR5 and (C) TLR4 expression in the various tissues are represented by the graphs. The relative density of proteins was normalized with β-actin. The data were based on the three independent experiments (*n* = 4 mice per each group). Differences were analyzed by Mann–Whitney *U*-test. *P <* *0.05; ***P <* *0.001; and ns, not significant compared with the tissues from the young mice.

Because this macrophage model is limited for explaining total innate immune responses, we evaluated the TLR4/5 expression in several tissues, including the spleen, skin, and intestine from young and aged mice. Notably, the protein expression of TLR5, but not TLR4, was well expressed in the spleen, skin, and intestine from both young and aged mice (Fig.4A). We collected these tissues from additional young and aged mice and evaluated the protein expression levels of TLRs 4 and 5 by Western blot. The quantitative graph demonstrates that the TLR5 protein was remarkably sustained in the aged tissues, similar to the young tissues (Fig.4B), indicating that TLR5 expression and FlaB-dependent TLR5 activation may be functional in the innate immune systems of the aged mice. However, it was difficult to detect TLR4 protein expression in the tissues from the aged mice (Fig.4C).

### FlaB/PspA immunization induces Ig production and confers protection against subsequent *S. pneumoniae* infection in aged mice

**Fig 5:**
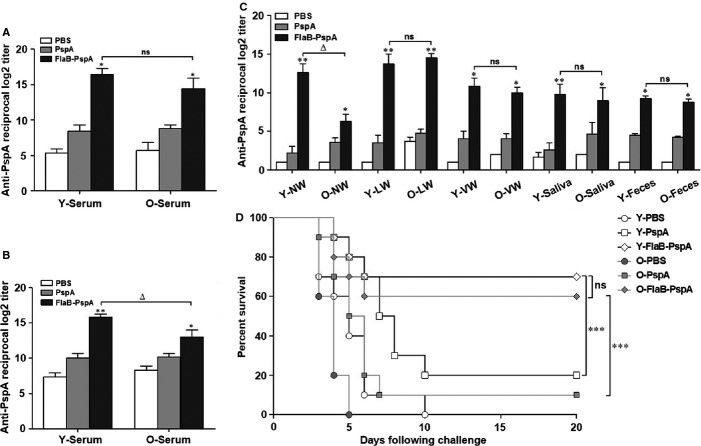
Antibody response against antigen-conjugated flagellin and the survival rate against *S. pneumonia* challenge in young and aged mice. After three rounds of intranasal immunization with FlaB-PspA or PspA in the young and aged mice, the sera and mucosal secretions were collected from the mice, and the PspA-specific antibody production was analyzed by ELISA. PspA-specific (A) IgG and (B) IgA responses in the serum from the young and aged mice were measured by ELISA. (C) PspA-specific IgA responses in various mucosal secretions were estimated by ELISA and are represented by the graph. To confirm the survival of (D) young and aged mice challenged with a 100X LD_50_ dose of *S. pneumoniae* D39, the mice were nasally immunized three times with 16 μL PBS (negative control), 2.5 μg PspA alone (control group), or 6.5 μg FlaB-PspA fusion protein and then challenged with *S. pneumoniae*. The data represent the mean ± SEM of (A-C) the PspA-specific antibody responses (*n* = 5 for each group) and (D) the survival rate of the young and aged mice (*n* = 10 mice per each group). The data were based on three independent experiments (Fig.5A–C) or one experiment (Fig.5D). Differences were analyzed by Mann–Whitney U-test (Fig.5A–C) and log-rank (Mantel–Cox test) (Fig.5D). *P <* *0.05; **P <* *0.01; ***P <* *0.001; compared with PspA alone in the young or aged mice, Δ, *P* < 0.05; ns, not significant compared with FlaB-PspA in the young and aged mice. NW, nasal wash; LW, lung wash; VW, vaginal wash.

Because our results demonstrate that TLR5 expression is well preserved in innate immune cells and various tissues from aged mice and that FlaB-dependent cytokines are significantly induced in aged macrophages, we determined whether FlaB enhances the innate immune responses through TLR5 activation in aged mice. To elucidate the FlaB-dependent immune activation in aged mice, we evaluated the effect of a vaccine against pneumococcal infections. We intranasally immunized young and aged mice with PspA or FlaB-PspA, as previously described (Nguyen *et al*., 2011). After three rounds of immunization, the PspA-specific antibody responses in both the serum and mucosal secretions were measured. In the serum sample of both young and old mice, the PspA-specific total IgG and IgA responses were higher in the FlaB-PspA group than PspA group (Fig.5A and B). In the other samples, with the exception of the nasal washes, the IgA response to FlaB-PspA immunization in the aged mice was significantly increased compared with immunization with PspA alone, similar to the results in the young mice (Fig.5C). These results indicate that the administration of FlaB-PspA induced effective systemic and mucosal immunity in the aged mice.

To determine whether FlaB may be used as a mucosal vaccine adjuvant against pneumococcal infection in aged mice, we examined the efficacy of the FlaB-PspA vaccine platform to induce protection from subsequent *S. pneumoniae* infection in the aged mice. When we infected the young and aged mice with *S. pneumoniae*, all of the aged mice died within 5 days, whereas all of the young mice survived (data not shown). This result confirmed that the aged mice had a high susceptibility to *S. pneumoniae* infection. Therefore, we administered either FlaB-PspA or PspA alone to the young and aged mice. Two weeks after the third administration of the vaccine, the mice were challenged intranasally with *S. pneumoniae*. Even though PspA alone could improve survival rate, however, FlaB-PspA was significantly effective in protecting against infection in young and old mice (Fig.5D). Interestingly, the aged mice vaccinated with FlaB-PspA exhibited a significantly increased survival rate of up to 60% compared with the mice immunized with PspA alone. The increased survival rate in aged mice had similarly effect in young mice. (Fig.5D). This finding supports the hypothesis that FlaB-dependent TLR5 activation may provide robust innate immunity in aged mice and that FlaB-PspA may be a successful vaccine adjuvant to pneumococcal infection in the elderly.

## Discussion

Because age-associated defects in the innate immune system are associated with the increased recruitment of phagocytic cells (Lord *et al*., 2001), we compared age-associated alterations in the innate immune system using a macrophage model by infectious stimuli that act through TLRs (Vabulas *et al*., 2002). Up to now, the results of human and mouse studies evaluating the effects of aging on TLR-dependent responses of monocytes have been conflicting. In aged (18–24 months old) and young (2–3 months old) mice, reverse transcriptase–polymerase chain reaction (RT-PCR) was performed to demonstrate decreased TLR1-9 expression (Renshaw *et al*., 2002). In addition, aged mice exhibit lower TLR4 protein levels and TLR4-dependent cytokine production than young mice do (Boehmer *et al*., 2004). The macrophages from aged mice exhibit no difference in TLR2 and TLR4 expression, but these receptors are dramatically decreased their response to agonist stimulation than those from young macrophages (Boehmer *et al*., 2005)

In our study, the increased number of macrophages might compensate for their reduced functions, including phagocytosis and cytokine production (Wang *et al*., 1995). Based on TLR screening in macrophages from young and aged mice, we observed that whereas the expression and response of TLR4 and the expression and response of other TLRs were not induced, notably, TLR5 expression and FlaB-dependent TLR5 signaling were well preserved in the aged macrophages. Although the role of macrophages as antigen-presenting cells does not entirely explain their function in innate immunity, many previous aging-associated TLR studies have been performed in macrophages from aged mice (Renshaw *et al*., 2002; Chelvarajan *et al*., 2006). To evaluate the role of FlaB-dependent TLR5 activation, we determined the expression of TLR5 in various tissues (including the spleen, skin, and intestine) from young and aged mice with TLR4 expression. We found that the expression of TLR5, but not TLR4, was well preserved in the aged tissues, similar to the young tissues. This maintenance of TLR5 might be regulated by caveolin-1. In this study, we suggest a novel function of caveolin-1 in innate immune systems as a mediator of the TLR5 signaling cascade. Furthermore, caveolin-1 directly interacted with TLR5/Myd88 by FlaB treatment and enhanced the signaling cascade, including NF-kB translocation and pro-inflammatory cytokines production. Caveolin-1-dependent TLR5/MyD88 signaling was also repeated in macrophages from caveolin-1 or MyD88 KO mice compared with WT mice. These results suggest that caveolin-1 upregulation plays a crucial role in innate immunity via regulating of TLR5 signaling and during the aging process.

To elucidate the role of FlaB-dependent TLR5 in aged mice, we designed a vaccine model against pneumococcal infection. Recently, our colleagues proved that the recombinant FlaB-PspA fusion protein enhances cross-protective immunity against *S. pneumoniae* infection in a mouse model (Nguyen *et al*., 2011). Thus, to evaluate FlaB as a mucosal adjuvant in aged mice, we used the FlaB-PspA fusion protein as an antigen–adjuvant model compared with PspA alone. Notably, FlaB-PspA induced significantly better IgG and IgA responses in the serum from the young and aged mice than did PspA alone, suggesting that the preservation of TLR5 activation during aging successfully induce innate and adaptive immune responses in immune-suppressed, aged hosts.

Recent studies have revealed that specific TLR agonists can enhance the protective efficacy of these vaccines against viral or bacterial infections in aged mice. Nasal delivery of a combined DNA adjuvant offers an attractive possibility for protection against *S. pneumoniae* in aged mice (Fukuyama *et al*., 2011). Another study suggested that a vaccine that links viral epitopes to flagellin exhibits protective efficacy against influenza infection in aged mice and the elderly (Leng *et al*., 2011; Taylor *et al*., 2011). In the present investigation, we also demonstrated robust mucosal adjuvant activities using a genetically engineered FlaB-PspA fusion protein against *S. pneumoniae* infection in aged mice. Moreover, a recent paper reported that the elderly may also display elevated TLR5 levels, leading to the induction of IL-8 and TNF-α production (Qian *et al*., 2012). These reports, including our results strongly suggest that TLR5 may provide a critical mechanism for enhancing the immune responsiveness in older individuals. Furthermore, we demonstrated the molecular mechanism of preserving TLR5 expression and signaling via caveolin-1 upregulation in innate immunosenescence.

Our results prove the molecular mechanism of well-maintained TLR5 expression and signaling via caveolin-1 upregulation in old macrophages and provide a new model for developing a vaccine adjuvant against pneumococcal infection in the elderly.

## Materials and methods

### Animals and isolation of peritoneal macrophages

Young female C57BL/6J mice (8–10 weeks old) were purchased from The Jackson Laboratory (Bar Harbor, ME, USA). Aged female C57BL/6J mice (24 months old) were provided from the Korea Basic Science Institute (Daejeon, Korea). Caveolin-1 knockout mice (backcrossed to C57BL/6J background for 10 generations) were purchased from The Jackson Laboratory. These mice were inbred by homozygotes mating. The mice were used at 8–10 weeks old for our experiments. MyD88 knockout mice (backcrossed to C57BL/6J background, 8–10 weeks old) were kindly provided by Prof. Rhee. All of the mice were maintained in a pathogen-free animal facility at the Clinical Vaccine R&D Center of Chonnnam National University. All of the mouse procedures were conducted in accordance with the guidelines of the Animal Care and Use Committee of Chonnam National University.

The isolation of peritoneal macrophages is described in Data S1 (Supporting information).

### Analysis of phagocytosis and pro-inflammatory cytokine

To analyze the phagocytic ability of the macrophages, we used *Salmonella typhimurium* (SL1344). The bacterial culture and phagocytosis are described in Data S1 (Supporting information). For measurement of pro-inflammatory cytokine levels, macrophages were stimulated with *Salmonella* protein (Sal-P) extracts, E. coli O127:B8), and *Vibrio vulnificus* FlaB (Vv-FlaB) recombinant proteins; after 12 h, the culture supernatants were collected for ELISA. Details are described in Data S1 (Supporting information).

### Biochemical analysis

Total RNA or proteins were extracted from the peritoneal macrophages of young or aged mice and analyzed by RT–PCR or Western blotting. For downregulation or overexpression of caveolin-1, we used lentivirus (LV)-carrying siRNA-cav-1(shLenti1.1-cav1) or RFP-conjugated full length of caveolin-1 genes (Lenti H1.4-cav1/RFP). Caveolae-rich membrane fractions were performed essentially as previously described (Cho *et al*., 2004). Nuclear and cytoplasmic fractions were practiced using Nuclear Extract Kit from Active Motif (Carlsbad, CA, USA). All details are available in Data S1 (Supporting information).

### Immunofluorescence and immunoprecipitation

Cells were grown on glass coverslips, stained with specific antibodies, analyzed by a Zeiss LSM 510 confocal laser scanning microscope, and then analyzed by Zeiss LSM 510 confocal software (Carl Zeiss Microimaging, Inc., Jena, Germany). For immunoprecipitation, the cell lysates were collected after stimulation with Vv-FlaB for 12 h and then incubated with a mixture of protein G-Sepharose beads (Santa Cruz Biotechnology, Dallas, USA) and specific antibodies. The beads were isolated and detected by Western blotting using anti-Cav1, anti-TLR5, and anti-MyD88 antibodies. Details are available in Data S1 (Supporting information).

### Analysis of Immunoglobulin (Ig) production

Purified PspA (surface protein A of *S. pneumoniae*) as an antigen and the recombinant FlaB-PspA fusion protein were provided by Prof. Rhee’s group. The young and aged mice were intranasally treated three times per 2 weeks with 2.5 μg PspA and 6.5 μg recombinant fusion FlaB-PspA or with 16 μL PBS only (as a control). Two weeks after the third immunization, all samples were collected from the mice in each group (*n* = 10) to determine the PspA-specific IgG and IgA production. ELISAs were performed as previously described (Lee *et al*., 2006). After coating the plates with 1 μg PspA, each sample was incubated and washed. The IgG or IgA was detected by adding 50 μL TMD (3, 3′, 5, 5′-tetramethylbenzidine) substrate solution (BD Bioscience, San Diego, CA, USA). The absorbance was read on a microplate reader (Molecular Devices Corp., Menlo, CA, USA) at 450 nm. The titer represents the reciprocal of the dilution that yielded an optical density of 0.1 at 450 nm.

### *S. pneumonia* culture and infection

The preparation and infection *S. pneumoniae* were previously described (Nguyen *et al*., 2011). For the challenge study, the LD_50_ of *S. pneumonia* D39 strain was determined using 8-week-old SPF female C57BL/6J mice. The LD_50_ was calculated using the Reed and Muench method. Two weeks after the final immunization, 20 μL of the 100-fold LD_50_ dose of *S. pneumonia* D39 was used to challenge the young and aged mice. The mice were closely observed for 20 days and monitored for survival. The survival rates were determined using the Kaplan–Meier method (Kaplan & Meier, 1958).

### Statistical analysis

The statistical analysis was performed using prism 5 software (GraphPad, Inc., San Diego, CA, USA). Differences between the experimental groups were analyzed by Mann–Whitney *U*-test. Survival curves were analyzed with log-rank test (Mantel–Cox test) and were considered significant for *P *< 0.05. The data are shown of at least three independent experiments that represented as the mean ± SEM except survival data.

## Supporting Information

**Fig. S1** The interaction of TLR5 and caveolin-1 in macrophages from MyD88^−/−^ mice. Peritoneal macrophages from WT and MyD88^−/−^ mice were stimulated with Vv-FlaB. (A) The protein expression levels were analyzed by Western blotting with anti-Cav1 and anti-TLR5 antibodies. (B) The interactions of Cav1 and TLR5 were analyzed by an immunoprecipitation assay. The cell lysates were immunoprecipitated with an anti-Cav1 antibody and immunoblotted with anti-TLR5 and anti-Cav1 antibodies. (C) After 12 h of stimulation with Vv-FlaB, the culture supernatants were collected and analyzed by ELISA for IL-6 cytokine secretion. The data are presented as the mean ± SEM from triplicate wells. Differences were considered statistically significant at *P* values. *****P *<* *0.01, compared with the peritoneal macrophages from wild-type mice. MyD88^−/−^, MyD88 knockout mice.
