# Supplementary material for: Flagellin-dependent TLR5/caveolin-1 as a promising immune activator in immunosenescence
Source: Aging Cell. 2015 Jul 30;14(5):907–15. doi: 10.1111/acel.12383 (PMC4568978; doi:10.1111/acel.12383)
Supplement: Supplementary file 2 [file acel0014-0907-sd2.doc]

**Supporting Information**

**SI Materials and Methods**

**Animals**

Young female C57BL/6J mice (8-10 weeks old) and caveolin-1 knockout mice were purchased from The Jackson Laboratory (Bar Harbor, ME, USA). Aged female C57BL/6J mice (24 months old) were provided from the Korea Basic Science Institute (Daejeon, Korea). MyD88 knockout mice were kindly provided by Prof. Rhee. All of the mice were maintained in a pathogen-free animal facility at the Clinical Vaccine R&D Center of Chonnam National University. All of the mouse procedures were conducted in accordance with the guidelines of the Animal Care and Use Committee of Chonnam National University.

**Isolation of macrophages from the mouse peritoneum**

Peritoneal macrophages were isolated from the peritoneum of the young and aged mice. To harvest the macrophages, peritoneal lavage was performed with 10 mL phosphate-buffered saline (PBS) containing 10% fetal bovine serum (FBS, Gibco-BRL, Grand Island, NY, USA). The isolated peritoneal cells were seeded into cell culture dishes for 12 hours. After being washed, the adherent cells were purified and cultured in RPMI 1640 medium (Gibco-BRL) supplemented with 10% FBS and 1% antibiotics.

**Phagocytosis assay**

To analyze the phagocytic ability of the macrophages, we used *S. typhimurium* (SL1344). Cells were seeded (1 × 10^5^ cells) in 96-well plates in RPMI 1640 medium and infected with *S. typhimurium* (multiplicity of infection 1:10). The cells were allowed to phagocytose for 30 minutes and were then washed three times with PBS. Fresh medium containing gentamicin (10 μg/mL) was added, and the cells were incubated for an additional 30 minutes to kill any residual extracellular bacteria. The cells were then lysed in PBS containing 0.05% Triton X-100. The lysates were plated onto LB agar plates and the colonies were subsequently counted. The assays were performed in triplicate.

**Measurement of pro-inflammatory cytokine levels**

*Salmonella* protein (Sal-P) was extracted from the bacteria using lysis buffer [50 mM Tris–HCl (pH 7.4), 1 mM ethylenediaminetetraacetic acid (EDTA) and 1 mM phenylmethylsulfonyl fluoride (PMSF)]. LPS (*E. coli* O127:B8) was purchased from Sigma Chemicals (St. Louis, MO, USA). *V.* *vulnificus* FlaB (Vv-FlaB) recombinant proteins were obtained from Prof. Rhee . Peritoneal macrophages were stimulated with Sal-P (10 μg/mL), LPS (5 μg/mL) and Vv-FlaB (100 ng/mL). After stimulation for 12 hours, the cell culture supernatants were collected, and the secreted levels of IL-6, TNF-α and IL-1β were measured by ELISA (BD Biosciences, San Diego, CA, USA). The ELISAs were performed in accordance with the manufacturer’s instructions.

**RNA isolation and RT-PCR**

Total RNA was extracted from the peritoneal macrophages of young and aged mice using the QIAzol lysis reagent (QIAGEN Sciences, Germantown, MD, USA) in accordance with the manufacturer’s instructions. The RNA was reverse-transcribed using the TaKaRa one-step RT-PCR kit (TaKaRa Bio Inc., Otsu, Shiga, Japan), and PCR amplification was performed. The sequences of the PCR primers used in this study are as follows: TLR4 sense, 5’-TAC CAT CTT CCC AAT GTT TC-3’ and antisense, 5’-GAC ACT TGG GAT GAC ACC TG-3’; TLR5 sense, 5’-TAC CAT CTT CCC AAT GTT TC-3’ and antisense, 5’-GAC ACT TGG GAT GAC ACC TG-3’; and β-actin sense, 5’-TGG AAT CCT GTG GCA TCC ATG AAA C-3’ and antisense, 5’-TAA AAC GCA GCT CAG TAA CAG TCC G-3. The 30 cycles of the PCR reaction were performed at 94°C for 30 s (denaturation), 58°C for 30 s (annealing) and 72°C for 30 s (extension) using a TaKaRa PCR Thermal Cycler. The PCR product was separated on agarose gels and then stained with ethidium bromide. For quantification of mRNA results in lineage range, we performed PCR with different cycle numbers to ensure that our amplification was at exponential phage not plateau phase. The DNA band density was quantified by Image J software (NIH, Maryland, USA).

**Western blot analysis**

Western blot analysis was performed as previously described . Briefly, total proteins from the peritoneal macrophages and tissues harvested from the young and old mice were extracted in lysis buffer with brief sonication. Protein samples were separated using sodium dodecyl sulfate-polyacrylamide gel electrophoresis and transferred onto polyvinylidene fluoride membranes. The membranes were incubated with anti-TLR5, anti-TLR4 (IMGENIX, San Diego, CA, USA), anti-caveolin-1 (BD Biosciences), anti-MyD88 (Santa Cruz Biotechnology, Delaware, CA, USA) and anti-β-actin (Santa Cruz Biotechnology) antibodies overnight in a cold room. The membranes were then incubated with peroxidase-conjugated anti-rabbit and mouse secondary antibodies (Santa Cruz Biotechnology) for 1 hour at room temperature (RT) and then visualized using an enhanced chemiluminescence detection kit (Amersham ECL Kit; GE Healthcare, Buckinghamshire, UK). Protein expression was analyzed with the Multi Gauge Software (Microsoft, Redmond, Washington, USA).

**Infection with Lentivirus**

Lentiviruses (LVs) expressing siRNA-cav-1 (shLenti1.1-cav1) or full-length caveolin-1 genes (Lenti H1.4-cav1/RFP) were manufactured by Macrogen, Co. (Seoul, Korea). The LV infection was performed according to the manufacturer’s method. Briefly, peritoneal macrophages were seeded (1 x 10^5^ cells/96-well plates) and infected with 1 mL of LV for 8 hours. After incubation, the cells were supplied with growth medium containing 10% FBS and were harvested 48 or 72 hours later for further assays.

**Isolation of caveolae-rich membrane fractions**

**Caveolae-rich membrane fractions** were performed essentially as previously described . Briefly, young and aged macrophages were scraped into 3 mL of 500 mM sodium carbonate (pH 11.0). Lysis steps were performed using a loosely fitting Dounce homogenizer (30 strokes), a Polytron homogenizer (three 15 sec bursts; output, 50 sec), and a sonicator (three 10 sec bursts; output 30 sec). The cell lysates were then adjusted to 40% sucrose. Discontinuous sucrose gradients [5-30% (w/v)] were formed above, with both gradients in MBS containing 250 mM sodium carbonate, and then were centrifuged at 39,000 rpm (200,000 × *g*) for 18 hours in an SW41 rotor (Beckman Instruments; Brea, CA, USA). From the top of each gradient, 1-12 mL fractions were carefully collected. The protein localizations of the gradient fractions were determined by Western blotting using anti-caveolin-1,anti-TLR5, and anti-TLR4 antibodies.

**Nuclear and cytoplasmic fraction**

Nuclear and cytoplasmic fractions were practiced by using Nuclear Extract Kit from Active Motif (Carlsbad, CA, USA) in accordance with the manufacturer’s instructions. Briefly, cells were washed with cold PBS/Phosphate inhibitor and detached. The cells were suspended with 1X Hypotonic Buffer and centrifuged for 30 seconds at 14,000 x g. The supernatant (cytoplasmic fraction) was transferred into new tube. Next, nuclear pellet was resuspended with complete lysis buffer and incubated for 30 minutes on ice. The suspended lysate was centrifuged 10 minutes at 14,000 x g and harvested the supernatant (nuclear fraction). The nuclear and cytoplasmic fractions were determined by Western blotting using anti-Lamin B (for nuclear protein, Abcam) and α-tubulin (for cytoplasmic protein, Santa Cruz Biotechnology).

**Immunofluorescence staining**

Cells were grown on glass coverslips for 1 day and fixed in 4% paraformaldehyde. After being washed with PBS, the cells were permeabilized with 0.1% Triton X-100 in PBS for 10 minutes. After being washed, the samples were blocked with 1% bovine serum albumin in PBS for 1 hour at RT and were incubated with an anti-TLR5 antibody (or anti-caveolin-1 antibody or anti-NF-κB antibody, Santa Cruz Biotechnology) at 4°C for overnight. After being washed, the samples were incubated with Alexa Fluor 488 (or Alexa Fluor 555, Invitrogen Co., Eugene, OR, USA) at RT for 1 hour. For staining of DNA, the samples were treated with DAPI at RT for 1 hour and then were mounted with a mounting medium. Fluorescence was visualized by a Zeiss LSM 510 confocal laser scanning microscopes and then analyzed by the Zeiss LSM 510 confocal software (Carl Zeiss Microimaging, Inc.; Jena, Germany).

**Immunoprecipitation**

Cells were stimulated with Vv-FlaB for 12 hours. The cells were lysed by sonication in lysis buffer and were centrifuged at 13,000 rpm for 10 minutes. Protein lysates were incubated with protein G-Sepharose beads (Santa Cruz Biotechnology) for 1 hour at 4°C and subsequently treated with anti-TLR5, anti-caveolin-1, or anti-MyD88 antibodies (in the protein lysates) at 4°C overnight. The beads were washed with lysis buffer several times and dissolved with a protein sample buffer. After centrifugation at 10,000 rpm for 5 minutes, the supernatants were collected, separated by SDS-PAGE, and then detected by Western blotting using anti-caveolin-1, anti-TLR5, and anti-MyD88 antibodies.

**Analysis of Immunoglobulin (Ig) production**

Purified PspA (surface protein A of *S. pneumoniae*) as an antigen and the recombinant FlaB-PspA fusion protein were provided by Prof. Rhee’s group. The young and aged mice were intranasally treated three times per 2 weeks with 2.5 μg PspA and 6.5 μg recombinant fusion FlaB-PspA or with 16 μL PBS only (as a control). Two weeks after the third immunization, all samples were collected from the mice in each group (n=10) to determine the PspA-specific IgG and IgA production. ELISAs were performed as previously described . After coating the plates with 1 μg PspA, each sample was incubated and washed. The IgG or IgA was detected by adding 50 μL TMD (3, 3’, 5, 5’-tetramethylbenzidine) substrate solution (BD Bioscience, San Diego, CA, USA). The absorbance was read on a microplate reader (Molecular Devices Corp., Menlo, CA, USA) at 450 nm. The titer represents the reciprocal of the dilution that yielded an optical density of o.1 at 450 nm.

***S. pneumonia* culture and infection**

The preparation and infection *S. pneumoniae* were previously described . For the challenge study, the LD_50_ of *S. pneumonia* D39 strain was determined using 8-week-old SPF female C57BL/6J mice. The LD_50_ was calculated using the Reed and Muench method . Two weeks after the final immunization, 20 μL of the 100-fold LD_50_ dose of *S. pneumonia* D39 was used to challenge the young and aged mice. The mice were closely observed for 20 days and monitored for survival. The survival rates were determined using the Kaplan-Meier method .

**Statistical Analysis**

The statistical analysis was performed using Prism 5 software (GraphPad, Inc., San Diego, CA, USA). Differences between the experimental groups were analyzed by Mann Whitney test. Survival curves were analyzed with Log-rank test (Mantel-Cox Test) and were considered significant for *p* values <0.05. The data are shown of at least three independent experiments that represented as the mean ± SEM except survival data.

**Supplementary Figure Legend**

**Figure S1. The interaction of TLR5 and caveolin-1 in macrophages from MyD88^-/-^ mice.** Peritoneal macrophages from WT and MyD88^-/-^ mice were stimulated with Vv-FlaB. (A) The protein expression levels were analyzed by Western blotting with anti-Cav1 and anti-TLR5 antibodies. (B) The interactions of Cav1 and TLR5 were analyzed by an immunoprecipitation assay. The cell lysates were immunoprecipitated with an anti-Cav1 antibody and immunoblotted with anti-TLR5 and anti-Cav1 antibodies. (C) After 12 hours of stimulation with Vv-FlaB, the culture supernatants were collected and analyzed by ELISA for IL-6 cytokine secretion. The data are presented as the mean ± SEM from triplicate wells. Differences were considered statistically significant at *P* values. **, *p* <0.01, compared with the peritoneal macrophages from wild-type mice. MyD88^-/-^, MyD88 knockout mice.

**References**

Cho KA, Ryu SJ, Oh YS, Park JH, Lee JW, Kim HP, Kim KT, Jang IS, Park SC (2004). Morphological adjustment of senescent cells by modulating caveolin-1 status. *J Biol Chem*. **279**, 42270-42278.

Kaplan EL, Meier P (1958). Nonparametric Estimation from Incomplete Observations. *Journal of the American Statistical Association*. **53**, 457-481.

Lee SE, Kim SY, Jeong BC, Kim YR, Bae SJ, Ahn OS, Lee JJ, Song HC, Kim JM, Choy HE, Chung SS, Kweon MN, Rhee JH (2006). A bacterial flagellin, Vibrio vulnificus FlaB, has a strong mucosal adjuvant activity to induce protective immunity. *Infect Immun*. **74**, 694-702.

Lim JS, Choy HE, Park SC, Han JM, Jang IS, Cho KA (2010). Caveolae-mediated entry of Salmonella typhimurium into senescent nonphagocytotic host cells. *Aging Cell*. **9**, 243-251.

Nguyen CT, Kim SY, Kim MS, Lee SE, Rhee JH (2011). Intranasal immunization with recombinant PspA fused with a flagellin enhances cross-protective immunity against Streptococcus pneumoniae infection in mice. *Vaccine*. **29**, 5731-5739.

Reed LJ, Muench H (1938). A simple method of estimating the fifty percent end points. *Am. J. Epidemiol.* **27**, 493-497.
